# The application of diffusion tensor imaging in patients with mild cognitive impairment: a systematic review and meta-analysis

**DOI:** 10.3389/fneur.2025.1467578

**Published:** 2025-04-23

**Authors:** Xinle Zhao, Mengyue You, Wenyu Ren, Lixin Ji, Yongbo Liu, Meng Lu

**Affiliations:** ^1^Henan University of Chinese Medicine, Zhengzhou, China; ^2^The Third Clinical School of Henan University of Chinese Medicine, Zhengzhou, China; ^3^Luoyang Branch of Dongzhimen Hospital Affiliated to Beijing University of Chinese Medicine, Luoyang Hospital of TCM, Luoyang, China

**Keywords:** mild cognitive impairment, diffusion tensor imaging, Alzheimer’s disease, elder, white matter

## Abstract

**Objective:**

To systematically evaluate the diagnostic value of diffusion tensor imaging (DTI) for mild cognitive impairment (MCI) based on Meta-analysis.

**Materials and methods:**

Databases including PubMed, Web of Science, Cochrane Library, Embase, China National Knowledge Infrastructure (CNKI), Wanfang and VIP database were searched for literature on the use of DTI in studying MCI. The search was conducted from the inception of each database up to February 20, 2024. Literature was screened based on predefined inclusion and exclusion criteria, relevant data were extracted, and the quality of the included studies was assessed using the QUADAS-2 tool. Heterogeneity was evaluated using the Q-test and *I^2^* statistics. Fractional anisotropy (FA) values for different brain regions (frontal lobe, parietal lobe, temporal lobe, occipital lobe, fornix, hippocampus, parahippocampal gyrus, posterior cingulum, posterior limb of the internal capsule, uncinate fasciculus, inferior fronto-occipital fasciculus, superior longitudinal fasciculus, inferior longitudinal fasciculus, genu and splenium of the corpus callosum) were extracted from the MCI and normal control (NC) groups. Meta-analysis software (Review Manager 5.4) was used to perform a pooled analysis of the eligible studies to obtain the weighted mean difference (WMD) and 95% confidence interval (95% CI).

**Results:**

A total of 76 studies were included (41 in English and 35 in Chinese). The overall pooled WMD and its 95% CI were −0.03 [−0.04, −0.03], with statistically significant differences in all brain regions except for the occipital lobe and the posterior limb of the internal capsule.

**Conclusion:**

DTI technology can identify microstructural damage in the brain white matter of MCI patients, which holds significant implications for early diagnosis and intervention.

## 1 Introduction

Mild cognitive impairment (MCI) is a transitional state between normal aging and dementia, characterized by objective evidence of cognitive decline, with a high likelihood of progressing to dementia. Therefore, it is essential to intervene and treat individuals in this transitional state to delay the progression of cognitive impairment and reduce the incidence of dementia (1). Diffusion tensor imaging (DTI) detects the diffusion of water molecules within tissues, revealing the microstructure and integrity of brain white matter fibers. Fractional anisotropy (FA) values are commonly used quantitative parameters in DTI examinations to analyze changes in white matter fibers. These values are crucial for identifying microstructural damage in the white matter of MCI patients (2, 3). Currently, numerous studies have been conducted globally on the use of DTI in diagnosing MCI. However, variations in results across these studies are due to various reasons. Therefore, this study systematically reviews the literature up to February 20, 2024, to evaluate the diagnostic value of DTI for MCI through meta-analysis. This analysis aims to provide objective evidence for selecting clinical examination methods and assessing diagnostic efficacy.

### 1.1 Literature search and selection

The literature search included both Chinese and English databases: PubMed, Web of Science, Cochrane Library, Embase, CNKI, Wanfang, and VIP databases. The Chinese search terms used were “轻度认知障碍” (mild cognitive impairment), “轻度认知功能损害” (mild cognitive disorders), “轻度认知功能损伤” (mild cognitive impairment), and “扩散张量成像” or “弥散张量成像” (diffusion tensor imaging). The English search terms included “mild cognitive impairment,” “mild cognitive disorders,” “magnetic resonance imaging,” and “diffusion tensor imaging.” The search covered the period from database inception to February 20, 2024.

### 1.2 Inclusion and exclusion criteria

Inclusion Criteria: In the literature screening process, we strictly adhered to the PRISMA guidelines. The inclusion criteria were as follows: (1) The selected studies were published in Chinese or English before February 20, 2024. (2) The study subjects were clinically diagnosed patients with MCI based on the Petersen criteria, with a normal control (NC) group consisting of healthy individuals matched by age and gender. (3) The MCI diagnosis was based on memory complaints lasting for more than 6 months, confirmed by an informant, with other cognitive functions remaining relatively intact. The Mini-Mental State Examination (MMSE) score was ≥24, and the Clinical Dementia Rating (CDR) was 0.5. (4) The participants did not meet the diagnostic criteria for dementia. (5) No significant impairment in activities of daily living (ADL) or any history of psychiatric or neurological disorders was present. (6) DTI was used to extract the FA values from specific brain regions (frontal lobe, parietal lobe, temporal lobe, occipital lobe, cingulum, hippocampus, parahippocampal gyrus, posterior cingulum, posterior limb of the internal capsule, uncinate fasciculus, inferior longitudinal fasciculus, superior longitudinal fasciculus, body and splenium of the corpus callosum). The data were reported as $\bar{x}\pm s$ . (7) The literature should explicitly state the magnetic field strength used 1.5T or 3.0T.

Exclusion Criteria: (1) Studies published in languages other than Chinese or English. (2) Studies where the data could not be accessed. (3) Studies involving subjects with other neurological disorders (e.g., stroke, Parkinson’s disease) or psychiatric conditions (e.g., depression) that could potentially affect memory function. (4) Unpublished or duplicate studies. (5) Case reports, guidelines, reviews, and animal studies.

### 1.3 Literature screening process and data extraction

Two researchers independently conducted quality assessment using the revised QUADAS-2 tool in the Review Manager 5.4 software. The risk of bias was evaluated in four domains: (1) Case Selection: Assessed whether the cases were consecutively or randomly included in the study to avoid selection bias. (2) Index Test: Evaluated whether the interpretation of DTI data was conducted in a blinded manner. (3) Reference Standard: Required that the MCI diagnosis be based on internationally recognized standards (specifically, the Petersen criteria). (4) Flow and Timing: Assessed whether the follow-up time was adequate to reduce attrition bias. Each of these four domains was evaluated for clinical applicability and risk of bias, with results categorized as “low,” “high,” or “unclear” based on the relevant criteria included in the domain-specific questions. In case of disagreements, a third-party consultation was conducted to resolve the issue.

### 1.4 Quality assessment

Quality assessment was independently conducted by two researchers using the revised QUADAS-2 tool in Review Manager 5.4 software. The assessment focused on four domains: patient selection, index test, reference standard, and patient flow and timing. Each of the first three domains was evaluated for clinical applicability, and all four domains were assessed for bias risk. The criteria for each domain were classified as “low,” “high,” or “uncertain” based on relevant key questions. Any disagreements were resolved through consultation with a third researcher.

### 1.5 Statistical analysis

Statistical analyses were performed using Review Manager 5.4 software provided by the Cochrane Collaboration. Heterogeneity was assessed using *I^2^* statistics and the Q test. If *I^2^* < 50% and *p* > 0.1 indicated acceptable heterogeneity, and a fixed effects model (FEM) was used; otherwise, a random effects model (REM) was applied. The combined effect size was expressed as the weighted mean difference (WMD) with a 95% confidence interval (95% CI). *p* < 0.05 was considered statistically significant. For studies with ≥10 articles, publication bias was assessed using a funnel plot; with good symmetry indicating the absence of publication bias.

## 2 Results

### 2.1 Literature screening process and results

**Figure 1:**
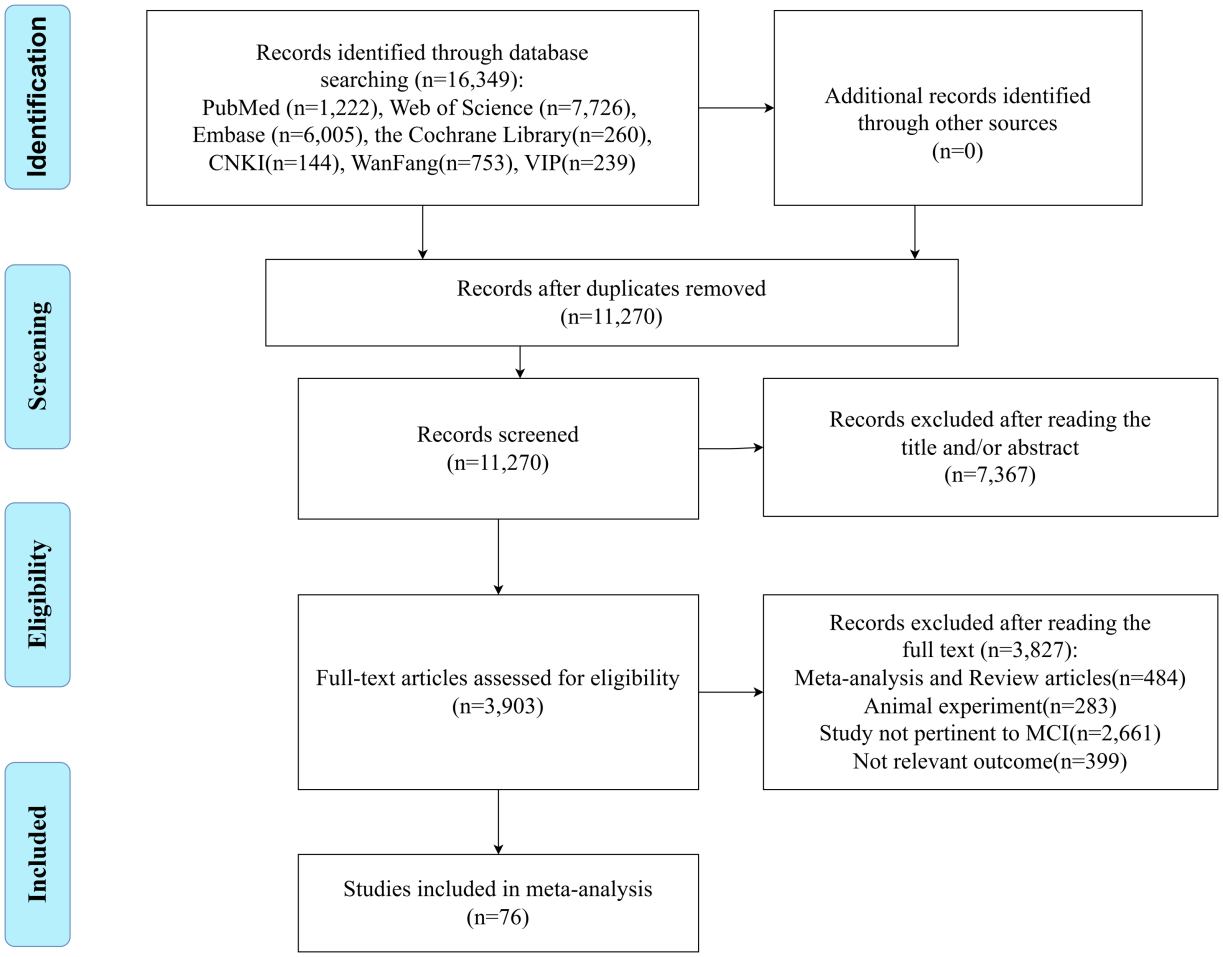
Literature screening process and results.

A total of 16,349 articles were retrieved, and after a stepwise screening process, 76 studies were included in the meta-analysis (41 in English and 35 in Chinese). The 76 studies included 1,973 MCI patients and 2,473 healthy controls. The literature screening process is depicted in Figure 1, and the basic characteristics of the included studies are summarized in Table 1.

**Table 1:** Basic characteristics of included studies.

Study	Country	Field strength	MCI (n)	MCI (age)	MCI (male/female)	NC (n)	NC (age)	NC (male/female)	Location	Technique data
Fellgiebel A, et al. 2004 (37)	Germany	1.5 T	14	68.2 ± 9.2	5/9	10	62 ± 6.8	7/3	F, CCG, CCS, O, P, T, H	FA
Fellgiebel A, et al. 2005 (38)	Germany	1.5 T	17	67.5 ± 8.9	11/6	21	67.7 ± 8.5	13/8	PC	FA
Gao GF 2006 (39)	China	3.0 T	23	70.3 ± 3.0	10/13	20	69.0 ± 2.9	9/11	F, T, P, O, H CCG, CCS, PC	FA
Huang J, et al. 2007 (40)	USA	1.5 T	8	74.8 ± 8.6	4/4	6	71.2 ± 5.7	2/4	F, T, P, O	FA
Müller MJ, et al. 2007 (41)	Germany	1.5 T	18	67.3 ± 8.7	11/7	18	66.9 ± 9.0	11/7	H	FA
Zhang Y, et al. 2007 (34)	USA	1.5 T	17	73.1 ± 7.4	9/8	18	71.6 ± 9.2	10/8	PC	FA
Cho H, et al. 2008 (25)	Korea	1.5 T	11	72.6 ± 7.3	5/6	11	70.6 ± 2.9	6/5	T, H, O, F, ICP, CCG, CCS, SLF, ILF, PC	FA
Fujie S, et al. 2008 (42)	USA	3.0 T	16	71.7 ± 7.1	4/12	16	70.9 ± 4.0	4/12	UF	FA
Shim YS, et al. 2008 (43)	Korea	1.5 T	21	72.8 ± 6.9	9/12	17	68.8 ± 3.6	8/9	T, H, O, P, F, CCG, CCS	FA
Ukmar M, et al. 2008 (44)	Italy	1.5 T	18	72.3 ± 10.5	10/8	15	59.5 ± 6.9	4/11	F, P, T, O, CCG, CCS	FA
Chen TF, et al. 2009 (45)	China	1.5 T	10	71.0 ± 9.1	7/3	20	70.1 ± 7.1	9/11	T, CCG, CCS	FA
Goldstein FC, et al. 2009 (46)	USA	3.0 T	14	71.5 ± 8.2	−/−	9	71.1 ± 7.4	−/−	T, F,	FA
Kiuchi K, et al. 2009 (47)	Japan	1.5 T	16	72.8 ± 9.1	11/5	16	71.9 ± 7.2	8/8	UF, PC	FA
Mielke MM, et al. 2009 (48)	USA	3 T	25	75.8 ± 5.3	18/7	35	74.3 ± 7.1	11/24	PC, FO	FA
Rogalski EJ, et al. 2009 (49)	USA	1.5 T	14	76.8 ± 7.0	4/10	14	73.6 ± 6.7	9/5	PH	FA
Chang C, et al. 2009 (50)	China	3 T	20	70.55 ± 6.55	8/12	20	71 ± 5.33	10/10	CCG, CCS, ICP, SLF, ILF, IFOF	FA
Chen H, et al. 2009 (51)	China	1.5 T	23	67.7 ± 7.0	12/11	26	67.5 ± 5.6	15/11	F, T, P, O, PC, PH CCG, CCS	FA
Cui JL, et al. 2009 (52)	China	3 T	32	59.21 ± 7.46	19/13	30	61.26 ± 5.71	14/16	SLF, ILF	FA
Liao J, et al. 2009 (53)	China	3.0 T	9	74.9 ± 2.8	7/2	11	74.8 ± 5.9	5/6	T, F, P, O, H, PH, PC, CCG, CCS	FA
Wu T, et al. 2009 (54)	China	1.5 T	15	66.0 ± 8.0	7/8	20	66.0 ± 5.0	9/11	F, P, O, T, CCG, CCS	FA
Choo IH, et al. 2010 (55)	Korea	3.0 T	19	71.6 ± 7.1	6/13	18	70.7 ± 5.2	6/12	PH, PC	FA
Pievani M, et al. 2010 (56)	Italy	1.5 T	19	68.5 ± 7.9	10/9	15	69.8 ± 6.0	6/9	FO, UF, IFOF, ILF, SLF	FA
Fu JL, et al. 2010 (57)	China	3 T	20	70.6 ± 6.7	8/12	20	71 ± 5.3	10/10	T, P, O, H, IFOF, CCG, CCS, SLF	FA
Ling RJ, et al. 2010 (58)	China	3.0 T	15	76.9 ± 5.6	9/6	21	76.4 ± 6.0	19/2	F, T, P, O, CCG, CCS	FA
Wang JH, et al. 2010 (59)	China	3.0 T	12	73.8 ± 5.4	5/7	12	72.6 ± 5.3	5/7	F, P, T, O, CCS, ICP,	FA
Liu Y, et al. 2011 (60)	USA	1.5 T	27	75.0 ± 2.0	15/12	19	75.0 ± 6.0	11/8	PH, UF, FO ILF, SLF, CCG	FA
Zhang YZ, et al. 2011 (61)	China	3 T	20	70.55 ± 6.65	8/12	20	71 ± 5.33	10/10	IFOF, SLF, CCG, CCS	FA
Bai F, et al. 2011 (62)	China	1.5 T	22	72 ± 4.4	11/11	22	70.2 ± 5.4	11/11	IFOF, CCG, CCS, PC, SLF	FA
Ji M, et al. 2011 (63)	China	3.0 T	50	74.5 ± 4.8	22/28	30	72.5 ± 5.2	12/18	F, P, O, T, CCG, CCS, PC, H, ICP	FA
Bozoki AC, et al. 2012 (64)	USA	3.0 T	23	70.8 ± 7.9	12/11	16	65.9 ± 8.5	6/10	FO	FA
Delano-Wood L, et al. 2012 (65)	USA	1.5 T	20	77.7 ± 6.6	12/8	20	78.3 ± 6.3	8/12	PC, CCG, CCS	FA
Thillainadesan S, et al. 2012 (66)	Australia	3.0 T	92	-	55/37	238	-	100/138	PC, CF	FA
Zhuang L, et al. 2012 (67)	Australia	3.0 T	76	80.6 ± 4.50	50/26	306	79.2 ± 4.4	88/218	FO	FA
Zimny A, et al. 2012 (68)	Poland	1.5 T	23	66.0 ± 9.4	7/16	15	69.0 ± 7.9	6/9	ILF, IFOF, CCG, CCS, ICP, SLF, PC	FA
Huang TT, et al. 2012 (69)	China	3.0 T	28	63.8 ± 7.7	8/20	28	62.8 ± 8.0	12/16	F, O, P, PC, CCG, CCS	FA
Zhao Q, et al. 2012 (70)	China	1.5 T	24	63.61 ± 9.44	12/12	24	61.44 ± 8.51	12/12	H	FA
Hong YJ, et al. 2013 (71)	Korea	1.5 T	20	70.5 ± 5.2	7/13	35	71.4 ± 5.7	17/18	H, PC	FA
Nowrangi MA, et al. 2013 (72)	USA	3 T	25	75.8 ± 5.3	18/7	25	74.3 ± 7.1	11/14	PC, FO	FA
Sachdev PS, et al. 2013 (73)	Australia	3 T	39	80.74 ± 5.29	24/15	155	79.08 ± 4.36	61/94	FO	FA
Sali D, et al. 2013 (74)	Greece	1.5 T	44	78.0 ± 8.0	18/26	25	67.0 ± 9.0	12/13	CCG, CCS, PC, SLF	FA
Stricker NH, et al. 2013 (75)	USA	1.5 T	32	68.5 ± 10.8	13/19	81	67.7 ± 9.1	30/51	PC, PH, T	FA
Ning WT, et al. 2013 (76)	China	3 T	15	65 ± 10.25	10/5	13	60.08 ± 7.09	5/8	T, F, H, PC, CCG, CCS	FA
Carter SF, et al. 2014 (77)	England	3.0 T	11	74.1 ± 6.4	9/2	11	69.6 ± 5.5	3/8	PC, UF, SLF IFOF, ILF	FA
Duffy SL, et al. 2014 (78)	Australia	3.0 T	30	68.1 ± 8.4	19/11	22	64.3 ± 8.7	8/14	CCG, CCS, SLF	FA
Fu JL, et al. 2014 (79)	China	3 T	41	70.57 ± 6.32	20/21	20	71 ± 5.33	10/10	F, T, P, O, H, CCG, CCS, IFOF, SLF	FA
Larroza A, et al. 2014 (80)	Spain	3.0 T	9	75.4 ± 6.2	5/4	8	77.1 ± 5.5	4/4	PC, UF	FA
Papma JM, et al. 2014 (81)	Netherlands	3 T	51	74.1 ± 4.9	37/14	23	70.9 ± 5	13/10	SLF	FA
Scrascia F, et al. 2014 (82)	Italy	1.5 T	12	74.3 ± 2.1	9/3	9	74.1 ± 2.4	3/6	F, CCG	FA
He L, et al. 2014 (83)	China	1.5 T	26	65.8 ± 6.0	15/11	26	66.5 ± 3.6	14/12	T, F, P, PH	FA
Hou MD, et al. 2014 (84)	China	3.0 T	16	66.9 ± 7.8	10/6	12	62.9 ± 8.7	6/6	CCG, CCS, ICP, SLF, ILF, IFOF	FA
Liu D, et al. 2014 (85)	China	3 T	83	69.4 ± 7.5	45/38	85	68.4 ± 6.3	44/41	FO, CCG, UF	FA
Ren QY, et al. 2014 (86)	China	1.5 T	18	66.3 ± 7.8	12/6	18	66.5 ± 3.7	10/8	PC, PH, ICP	FA
Ren QY, et al. 2014 (87)	China	1.5 T	18	66.3 ± 7.8	12/6	18	66.5 ± 3.7	10/8	T, F, P, CCG, CCS	FA
Wu J, et al. 2014 (88)	China	3.0 T	30	69.2 ± 8.1	17/13	31	67.9 ± 8.4	19/12	UF, IFOF	FA
Zou WY, et al. 2015 (89)	China	3 T	41	70.57 ± 6.32	20/21	20	71 ± 5.33	10/10	F, T, P, O, H, IFOF, SLF, CCG, CCS	FA
Cooley SA, et al. 2015 (90)	USA	3 T	25	61.6 ± 8.5	9/16	19	59.3 ± 7.6	8/11	O, T, P, F	FA
Hong YJ, et al. 2015 (91)	Korea	1.5 T	47	70.5 ± 5.17	28/19	47	70.6 ± 6.48	28/19	H, PC, CCG, CCS	FA
Kehoe EG, et al. 2015 (92)	Ireland	3 T	18	68.83 ± 7.71	9/9	22	68.86 ± 6.47	12/10	FO	FA
Nishioka C, et al. 2015 (93)	USA	3.0 T	30	71.1 ± 5.9	15/15	30	70.9 ± 5.4	15/15	CCS	FA
Nowrangi MA, et al. 2015 (94)	USA	3.0 T	22	75.3 ± 5.4	15/7	25	74.3 ± 7.1	11/14	F, P	FA
Wang L, et al. 2015 (95)	China	1.5 T	12	68.25 ± 7.85	3/9	15	63.8 ± 8.05	4/11	T, F, O, PH, CCG, CCS, ICP	FA
Chen YY, et al. 2016 (96)	China	3 T	34	67.45 ± 8.65	16/18	22	68.15 ± 7.17	13/9	F, P, T, O, CCG, CCS	FA
Li WP, et al. 2017 (97)	China	3 T	17	66 ± 11	11/6	24	70 ± 10	16/8	FO, ILF, SLF	FA
Zhou ZM, et al. 2017 (98)	China	1.5 T	11	71.5 ± 5.1	7/4	91	70.9 ± 3.9	51/40	FO, PH,	FA
Li MJ, et al. 2018 (99)	China	3 T	30	60.07 ± 11.46	20/10	20	56.15 ± 9.41	13/7	H, T, F, CCG, CCS	FA
Zheng C, et al. 2018 (100)	China	3 T	32	69.13 ± 0.98	10/22	49	69.1 ± 0.77	16/33	FO	FA
Park CH, et al. 2019 (101)	Korea	3 T	16	71.38 ± 8.61	9/7	14	66 ± 4.95	5/9	UF	FA
Lin CC, et al. 2019 (102)	China	3 T	15	66.13 ± 10.45	8/7	15	65.29 ± 10.27	8/7	T, P, O, F, PC, ICP, CCG, CCS	FA
Yu H, et al. 2019 (103)	China	3 T	61	67.3 ± 7.6	32/29	60	66.2 ± 7.9	34/26	F, T, P, O, H, IFOF, SLF, CCG, CCS	FA
Bigham B, et al. 2020 (104)	Iran	3 T	24	76 ± 8.6	12/12	24	75.3 ± 8.3	11/13	F, O, P, T	FA
Luo CM, et al. 2020 (105)	China	3 T	36	67.58 ± 6.792	15/21	43	61.91 ± 6.358	23/20	CCG, CCS, UF, IFOF, ILF, SLF	FA
Qian FD, et al. 2020 (106)	China	3 T	48	78.3 ± 2.3	28/20	48	78.5 ± 2.1	26/22	P, F, T, O, CCS, CCG	FA
Liu MX, et al. 2021 (107)	China	3 T	28	69.15 ± 7.31	10/18	20	65.57 ± 7.82	10/10	PC	FA
Zhang Y, et al. 2021 (108)	China	3 T	25	74.44 ± 8.00	15/10	20	69.85 ± 9.33	8/12	UF, IFOF, ILF, SLF	FA
Li XT, et al. 2022 (109)	China	3 T	13	64.8 ± 8.8	6/7	11	60.9 ± 11	7/4	PC, PH	FA
Feng TB, et al. 2023 (110)	China	3 T	37	63.27 ± 6.35	20/17	40	63.67 ± 6.28	19/21	F, P, O, CCG, CCS	FA

F, frontal lobe; P, parietal lobe; T, temporal lobe; IFOF, inferior fronto-occipital fasciculus; O, occipital lobe; PC, posterior cingulated fasciculus; H, hippocampus; PH, parahippocampal gyrus; ICP, posterior limb of the internal capsule; SLF, fasciculus longitudinal superior; ILF, fasciculus longitudinal inferior; FO, fornix; CCG, genu of corpus callosum; CCS, splenium of corpus callosum; UF, uncinatus fasciculus.

### 2.2 Quality assessment results

**Figure 2:**
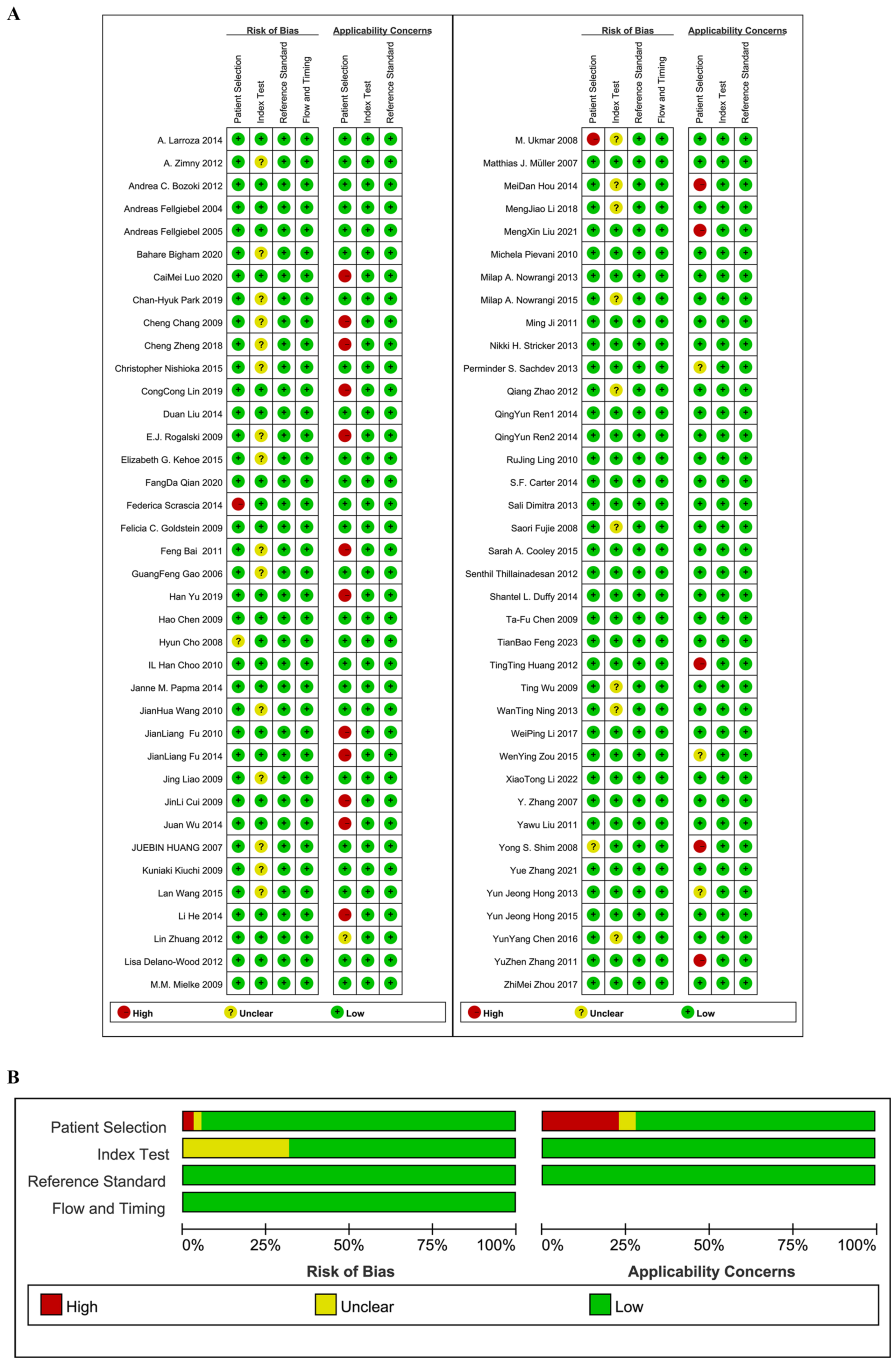
**(A)** Risk of bias assessments. **(B)** Risk of bias summary.

Quality assessment was conducted using the QUADAS-2 tool, and the results are shown in Figure 2. In the patient selection domain, 2 studies were rated as having an unclear risk of bias because it was not specified whether cases were randomly or consecutively enrolled; 2 studies were rated as having a high risk of bias due to non-random or non-consecutive case inclusion. Regarding clinical applicability, 4 studies were rated as having an unclear risk of bias because the inclusion of patients and their backgrounds did not clearly match the evaluation criteria, and 17 studies were rated as having a high risk of bias because the subjects were MCI subtypes. In the index test domain, 24 studies were rated as having an unclear risk of bias because it was not clear if the test interpretation was blinded to the reference standard results; the remaining studies were considered to have a low risk of bias and good clinical applicability.

### 2.3 FA value data analysis results

**Figure 3:**
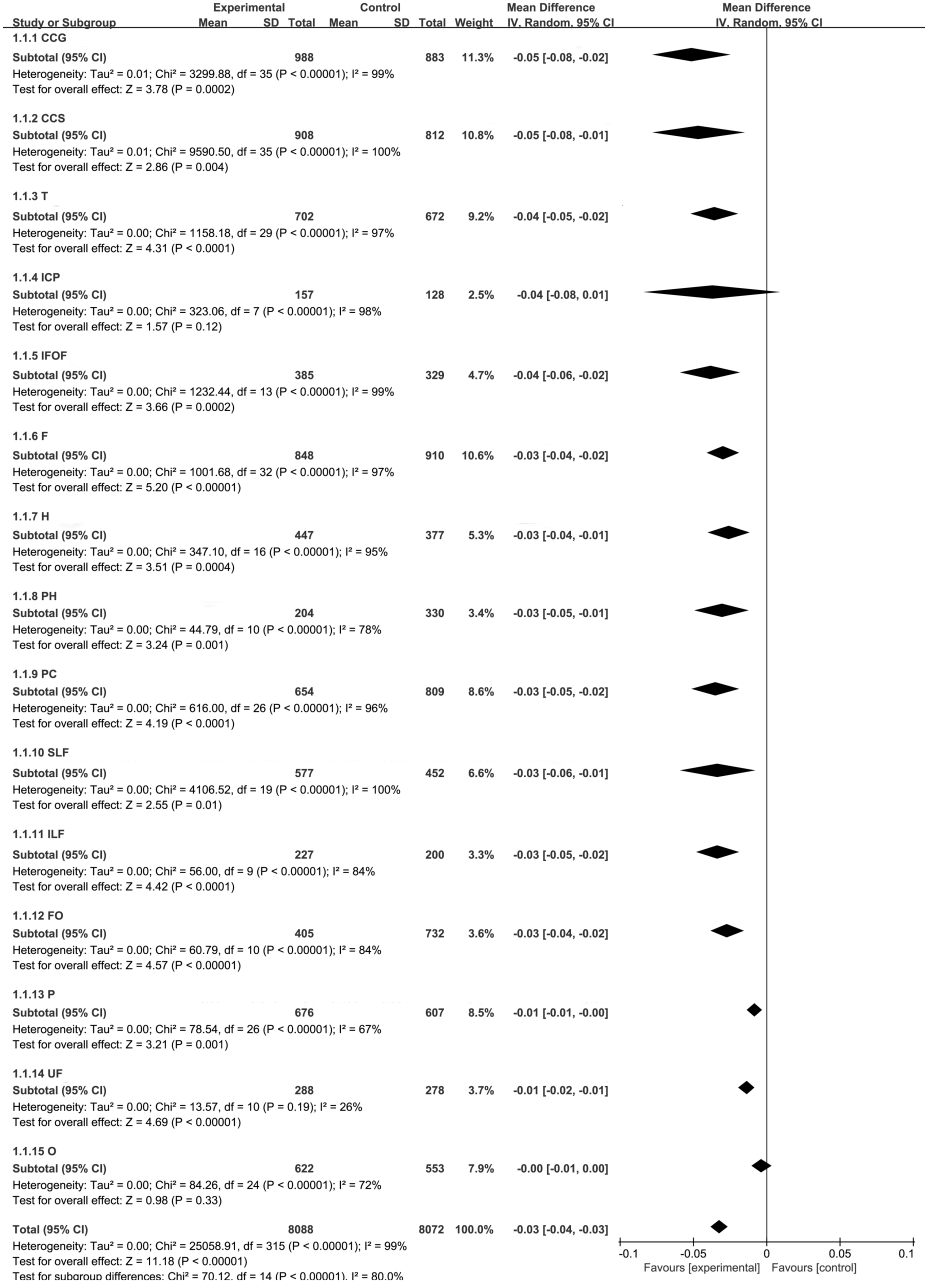
Integrated forest plot.

Among the 77 studies, a total of 318 comparisons were included, as shown in Figure 3. The comparisons include: 33 studies comparing FA values of the frontal lobe between MCI and NC groups. 27 studies comparing FA values of the parietal lobe. 30 studies comparing FA values of the temporal lobe. 25 studies comparing FA values of the occipital lobe. 17 studies comparing FA values of the hippocampus. 11 studies comparing FA values of the parahippocampal gyrus. 27 studies comparing FA values of the posterior cingulate gyrus. 8 studies comparing FA values of the posterior limb of the internal capsule. 20 studies comparing FA values of the superior longitudinal fasciculus. 10 studies comparing FA values of the inferior longitudinal fasciculus. 12 studies comparing FA values of the fornix. 37 studies comparing FA values of the genu of the corpus callosum. 36 studies comparing FA values of the splenium of the corpus callosum. 14 studies comparing FA values of the inferior fronto-occipital fasciculus. 11 studies comparing FA values of the uncinate fasciculus.

REM were used for analyzing the frontal lobe, parietal lobe, temporal lobe, occipital lobe, fornix, hippocampus, parahippocampal gyrus, posterior cingulum, posterior limb of the internal capsule, inferior fronto-occipital fasciculus, superior longitudinal fasciculus, inferior longitudinal fasciculus, genu and splenium of the corpus callosum. FEM was used for analyzing the uncinate fasciculus.

### 2.4 Meta-analysis results

#### 2.4.1 Overall results

The integrated forest plot is shown in Figure 3, summarizing 8,088 lesions and 8,072 normal controls. The overall WMD and its 95% confidence interval (CI) were −0.03 [−0.04–0.03], with a statistically significant difference (*p* < 0.05).

#### 2.4.2 Frontal lobe results

**Figure 4:**
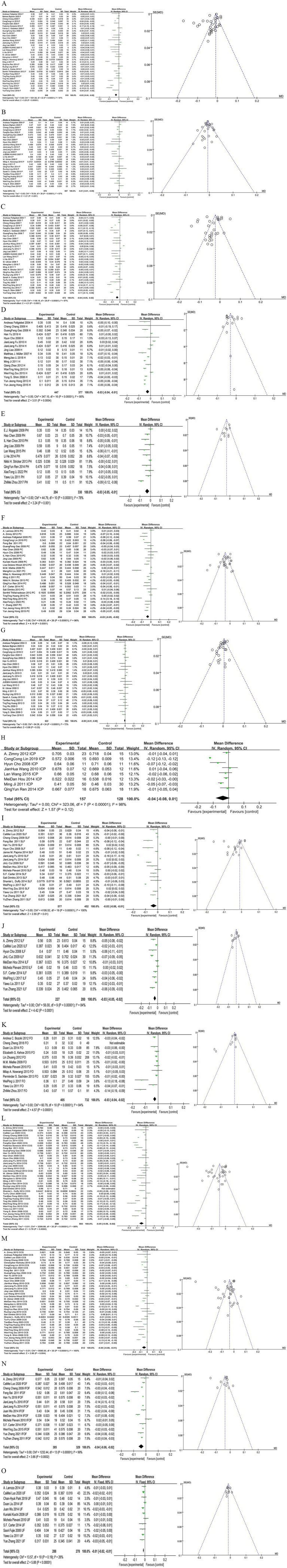
Forest map and funnel map of each brain area. **(A)** Forest map and funnel map of frontal lobe. **(B)** Forest map and funnel map of parietal lobe. **(C)** Forest map and funnel map of temporal lobe. **(D)** Forest map and funnel map of Hippocampus. **(E)** Forest map and funnel map of parahippocampal gyrus. **(F)** Forest map and funnel map of posterior cingulated fasciculus. **(G)** Forest map and funnel map of occipital lobe. **(H)** Forest map and funnel map of posterior limb of the internal capsule. **(I)** Forest map and funnel map of fasciculus longitudinal superior. **(J)** Forest map and funnel map of fasciculus longitudinal inferior. **(K)** Forest map and funnel map of fornix. **(L)** Forest map and funnel map of genu of corpus callosum. **(M)** Forest map and funnel map of splenium of corpus callosum. **(N)** Forest map and funnel map of inferior fronto-occipital fasciculus. **(O)** Forest map and funnel map of occipital lobe.

After pooling the frontal lobe fractional anisotropy (FA) values, we identified 848 lesions and 910 normal controls. The pooled WMD and its 95% CI were −0.03 [−0.04–0.02], with a statistically significant difference (*p* < 0.05). The funnel plot showed asymmetry, indicating potential publication bias (Figure 4A).

#### 2.4.3 Parietal lobe results

After pooling the parietal lobe FA values, we identified 676 lesions and 607 normal controls. The pooled WMD and its 95% CI were −0.01 [−0.01–0.00], with a statistically significant difference (*p* < 0.05). The funnel plot was symmetrical, indicating no potential publication bias (Figure 4B).

#### 2.4.4 Temporal lobe results

After pooling the temporal lobe FA values, we identified 702 lesions and 672 normal controls. The pooled WMD and its 95% CI were −0.04 [−0.05–0.02], with a statistically significant difference (*p* < 0.05). The funnel plot showed asymmetry, indicating potential publication bias (Figure 4C).

#### 2.4.5 Hippocampus results

After pooling the hippocampus FA values, we identified 447 lesions and 377 normal controls. The pooled WMD and its 95% CI were −0.03 [−0.04–0.01], with a statistically significant difference (*p* < 0.05). The funnel plot was symmetrical, indicating no potential publication bias (Figure 4D).

#### 2.4.6 Parahippocampal gyrus results

After pooling the parahippocampal gyrus FA values, we identified 204 lesions and 330 normal controls. The pooled WMD and its 95% CI were −0.03 [−0.05–0.01], with a statistically significant difference (*p* < 0.05). The funnel plot was symmetrical, indicating no potential publication bias (Figure 4E).

#### 2.4.7 Posterior cingulate gyrus results

After pooling the posterior cingulate gyrus FA values, we identified 654 lesions and 809 normal controls. The pooled WMD and its 95% CI were −0.03 [−0.05 to 0.02], with a statistically significant difference (*p* < 0.05). The funnel plot was symmetrical, indicating no potential publication bias (Figure 4F).

#### 2.4.8 Occipital lobe results

After pooling the occipital lobe FA values, we identified 622 lesions and 553 normal controls. The pooled WMD and its 95% CI were −0.00 [−0.01 to 0.00], with no statistically significant difference (*p* > 0.05). The funnel plot showed asymmetry, indicating potential publication bias (Figure 4G).

#### 2.4.9 Posterior limb of the internal capsule results

After pooling the posterior limb of the internal capsule FA values, we identified 157 lesions and 128 normal controls. The pooled WMD and its 95% CI were −0.04 [−0.08 0.01], with no statistically significant difference (*p* > 0.05). Due to the limited number of studies, publication bias was not assessed (Figure 4H).

#### 2.4.10 Superior longitudinal fasciculus results

After pooling the superior longitudinal fasciculus FA values, we identified 577 lesions and 452 normal controls. The pooled WMD and its 95% CI were −0.03 [−0.06–0.01], with a statistically significant difference (*p* < 0.05). The funnel plot showed asymmetry, indicating potential publication bias (Figure 4I).

#### 2.4.11 Inferior longitudinal fasciculus results

After pooling the inferior longitudinal fasciculus FA values, we identified 227 lesions and 200 normal controls. The pooled WMD and its 95% CI were −0.03 [−0.05–0.02], with a statistically significant difference (*p* < 0.05). The funnel plot was symmetrical, indicating no potential publication bias (Figure 4J).

#### 2.4.12 Fornix results

After pooling the fornix FA values, we identified 405 lesions and 732 normal controls. The pooled WMD and its 95% CI were −0.03 [−0.04–0.02], with a statistically significant difference (*p* < 0.05). The funnel plot showed asymmetry, indicating potential publication bias (Figure 4K).

#### 2.4.13 Genu of corpus callosum results

After pooling the genu of corpus callosum FA values, we identified 988 lesions and 883 normal controls. The pooled WMD and its 95% CI were −0.05 [−0.08–0.02], with a statistically significant difference (*p* < 0.05). The funnel plot was symmetrical, indicating no potential publication bias (Figure 4L).

#### 2.4.14 Splenium of corpus callosum results

After pooling the splenium of corpus callosum FA values, we identified 908 lesions and 812 normal controls. The pooled WMD and its 95% CI were −0.05 [−0.08–0.01], with a statistically significant difference (*p* < 0.05). The funnel plot was symmetrical, indicating no potential publication bias (Figure 4M).

#### 2.4.15 Inferior front-occipital fasciculus results

After pooling the FA values for the inferior fronto-occipital fasciculus, we identified 385 lesions and 329 normal controls. The pooled WMD and its 95% CI were −0.04 [−0.06, −0.02], indicating a statistically significant difference (*p* < 0.05). The funnel plot was symmetrical, indicating no potential publication bias (Figure 4N).

#### 2.4.16 Uncinate fasciculus results

After pooling the uncinate fasciculus FA values, we identified 288 lesions and 278 normal controls. The pooled WMD and its 95% CI were −0.01 [−0.02–0.01], with a statistically significant difference (*p* < 0.05). The funnel plot showed good symmetry, indicating no potential publication bias (Figure 4O).

## 3 Discussion

Mild cognitive impairment (MCI) is a transitional stage between normal aging and dementia, characterized by cognitive decline that exceeds normal age-related changes but does not meet the criteria for dementia (4). Alzheimer’s disease (AD) is the most common neurodegenerative disorder and the most prevalent type of dementia. The probability of MCI patients developing dementia within 1 year is 10–15%, and within 2 years, the probability is 40%, with the incidence rate increasing annually (5, 6). Studies have shown that some MCI patients experience cognitive improvement over time, and some even revert to normal cognitive function (7, 8). However, research by Roberts et al. (9) found that patients whose cognition returned to normal are more likely to progress to dementia than those who never had MCI. Therefore, early diagnosis and intervention in MCI patients are crucial.

Diffusion tensor imaging (DTI) is an advancement based on magnetic resonance diffusion-weighted imaging. It not only observes the movement speed of water molecules within tissues but also applies diffusion gradients in more than six directions to obtain anisotropic diffusion of water molecules within the plane. This forms images that trace the pathways of fibers, allowing for the non-invasive tracking of brain white matter fibers and reflecting their structural integrity and connectivity. DTI provides an objective basis for evaluating the pathophysiological changes in tissue structure, aiding in clinical diagnosis. In recent years, ex vivo micro-diffusion tensor imaging (micro-DTI) has demonstrated significant potential in neuroscience research. Studies have shown that micro-DTI can provide higher resolution and more precise information about microstructural changes, enabling fine quantification of FA changes in perforating pathways (10). This capability is particularly important for understanding the microstructural alterations of specific neural pathways. However, single DTI technology may not fully capture the complex pathophysiological processes of MCI. Quantitative Susceptibility Mapping (QSM) can quantify changes in iron deposition in the brain, particularly in the hippocampus and deep gray matter structures, where iron deposition is closely associated with the pathological features of MCI (11, 12). Additionally, QSM can assess myelin damage and venous oxygen saturation, providing supplementary information for the diagnosis of MCI (13, 14). The high resolution and quantitative capabilities of QSM enable it to offer more detailed tissue magnetic property information, thereby complementing DTI’s limitations in assessing the integrity of white matter fiber tracts. The combination of these two techniques can provide a more comprehensive understanding of brain structure and function, offering deeper insights into the underlying pathological processes of MCI and other neurodegenerative diseases, which is beneficial for the early diagnosis and intervention of MCI.

In studies on AD and MCI, the combination of DTI and functional magnetic resonance imaging (fMRI) with machine learning and deep learning techniques has significantly enhanced diagnostic accuracy. The diffusion-based graph contrast learning method (DGCL), through diffusion processes and graph contrastive learning, strengthens the consistency of brain networks, effectively mitigating the impact of individual differences on diagnostic outcomes, thereby improving the diagnostic accuracy for AD and MCI (15). The Decoupled Generative Adversarial Network (DecGAN) decomposes brain networks into sparse subgraphs and complementary graphs through a decoupling module. It utilizes an adversarial strategy to guide the decoupling module in extracting features more relevant to AD. By encoding the detected neural circuits using hypergraph data, DecGAN significantly enhances the diagnostic accuracy of AD (16). The model based on prior-guided adversarial learning and hypergraphs (PALH) guides multimodal representation learning by estimating the prior distribution of anatomical knowledge. It utilizes adversarial strategies to reduce the discrepancy between representation distributions. The hypergraph-aware network designed in this model effectively integrates the learned representations, establishing higher-order relationships both across and within modalities. This enhances the accuracy and reliability of abnormal connectivity prediction in AD (17). These studies are not only theoretically innovative but also provide significant practical insights, offering new perspectives and directions for the diagnosis and treatment of AD and MCI.

FA value is a key parameter used in DTI to quantify the directional diffusion of water molecules. It ranges from 0 to 1, with higher values indicating greater restriction of water molecule diffusion along a particular direction, typically reflecting the integrity of white matter tracts. This parameter is especially significant in many neurodegenerative diseases, particularly AD, as an early key biomarker. However, the interpretation of FA values must be considered within a complex biological context, as changes in FA may involve multiple mechanisms rather than just myelin damage. Firstly, a reduction in FA could be related to disruption of axonal membrane integrity, a decrease in axonal density, or dysfunction in axonal transport (18). Secondly, changes in the extracellular matrix due to glial cell proliferation or inflammatory responses might also reduce FA by increasing isotropic diffusion (19). Additionally, in areas of fiber crossing or branching (such as at the junction of the corpus callosum and the corona radiata), the natural multidirectionality of water molecule diffusion due to the presence of fibers in multiple directions can lead to a decrease in FA. This phenomenon is not indicative of pathological damage but rather reflects normal anatomical structures (20). It’s also worth noting that some studies suggest that a slight decrease in FA in specific brain regions may reflect fiber reorganization or compensatory repair processes, rather than purely white matter degeneration (21). Studies have shown that combining other diffusion metrics, such as mean diffusivity, radial diffusivity, and axial diffusivity, can provide a more comprehensive understanding of white matter integrity (22). Therefore, the clinical significance of the FA value should be interpreted in conjunction with the anatomical characteristics of the fiber tracts, the heterogeneity of the patient population, and multimodal imaging analyses.

The study found that patients with MCI exhibited significant reductions in the FA values of cortical-related brain regions, including the frontal lobe (WMD = −0.03, 95% CI: −0.04 to −0.02), hippocampus (WMD = −0.03, 95% CI: −0.04 to −0.01), and the splenium of the corpus callosum (WMD = −0.05, 95% CI: −0.05 to −0.02), suggesting impaired white matter integrity. These regions are closely associated with executive function, memory integration, and interhemispheric information transfer, potentially serving as biomarkers for early diagnosis. For instance, the reduced FA in the splenium of the corpus callosum is correlated with decreased functional connectivity in the bilateral frontal lobes, which may explain the executive control deficits observed in MCI patients. The WMD, 95% CI, heterogeneity (*I*^2^ value), and clinical significance of each brain region are shown in Table 2.

**Table 2:** Compilation of FA values in different brain regions and their clinical significance.

Brain region	WMD	95% CI	*I*^2^ value	Clinical significance
CCG	−0.05	−0.08 ~ −0.02	99%	Delayed information processing, impaired executive function
CCS	−0.05	−0.08 ~ −0.01	100%	Information integration disorder, poor language fluency
T	−0.04	−0.05 ~ −0.02	97%	Language and semantic memory impairment
ICP	−0.04	−0.08 ~ 0.01	98%	Motor control impairment, language function damage (post-injury)
IFOF	−0.04	−0.06 ~ −0.02	99%	Visual–spatial processing disorder, object recognition difficulty
F	−0.03	−0.04 ~ −0.02	97%	Decline in executive function, working memory impairment, attention deficits
H	−0.03	−0.04 ~ −0.01	95%	Memory decline, learning ability reduction
PH	−0.03	−0.05 ~ −0.01	78%	Spatial navigation and episodic memory abnormalities
PC	−0.03	−0.05 ~ −0.02	96%	Episodic memory decline, default network damage
SLF	−0.03	−0.06 ~ −0.01	100%	Visual spatial processing and attention deficits, goal-directed processing disorder
ILF	−0.03	−0.05 ~ −0.02	84%	Visuospatial memory and attention abnormalities
FO	−0.03	−0.04 ~ −0.02	84%	Memory decline, emotional fluctuations
P	−0.01	−0.01 ~ −0.00	67%	Spatial perception, spatial attention disorder
UF	−0.01	−0.02 ~ −0.01	26%	Emotional regulation disorder, social function impairment
O	0.00	−0.01 ~ 0.00	72%	Visual processing impairments, visuospatial processing abnormalities (post-injury)

The frontal and parietal lobes play significant roles in executive control functions. Zhao et al. (23) found that the functional connectivity of the frontal–parietal network is lower in AD patients, which is closely related to the decline in executive control functions observed in these patients (24). This may serve as a potential non-invasive biomarker for early diagnosis of AD. The superior and inferior longitudinal fasciculi connect the frontal and parietal lobes, transmitting sensory, visual, auditory, and proprioceptive information from the back of the brain to the front. These fiber tracts are crucial for memory, attention, and executive functions (25). The hippocampus is considered a critical brain region for memory and cognitive functions. The fornix, carrying most of its axons from the hippocampal output fibers, is an essential part of the hippocampal memory circuit (26). A reduction in the functional connectivity of the hippocampus is significantly associated with the disruption of fornix integrity (27). Amnestic MCI may predominantly affect brain regions associated with memory, such as the hippocampus and fornix, whereas non-amnestic MCI may show more pronounced reductions in FA values in brain areas related to executive function and language, such as the frontal and parietal lobes. The inferior fronto-occipital fasciculus connects the inferior dorsal frontal lobe, temporal lobe, and occipital lobe, playing a vital role in visual–spatial processing, object recognition, and memory. Compared to MCI patients, the effect size of the inferior fronto-occipital fasciculus is reduced in AD patients, marking it as an important site of white matter lesions (28). The corpus callosum, the main commissural fiber bundle connecting the two cerebral hemispheres, is crucial for integrating sensory and motor functions (29). Damage to the integrity of this part will reduce functional connectivity between the hemispheres, inducing cognitive impairment (25). Previous studies (30–32) have shown that cognitive functions are related to the cingulum and uncinate fasciculus. The cingulum is essential for episodic memory, while the uncinate fasciculus is associated with verbal memory, visual attention, verbal abstraction, and immediate recall cognitive functions.

In the studies included in this paper, most research focused on the left hemisphere of the brain and showed statistical significance. McHugh et al. (33) found that the dominant hemisphere in humans is often the left hemisphere, resulting in functional asymmetry between the brain hemispheres. The right hippocampus is more closely associated with spatial information, whereas most MCI patients primarily exhibit a decline in cognitive memory functions. Consequently, changes in the left hippocampus are more pronounced compared to the right, with the left hippocampus being smaller in volume than the right. Zhang et al. (34) reported that the FA values of fiber circuits in the left hemisphere decreased more in MCI patients compared to normal controls. Therefore, if data from both hemispheres were available in the included studies, the left hemisphere data were uniformly selected.

**Figure 5:**
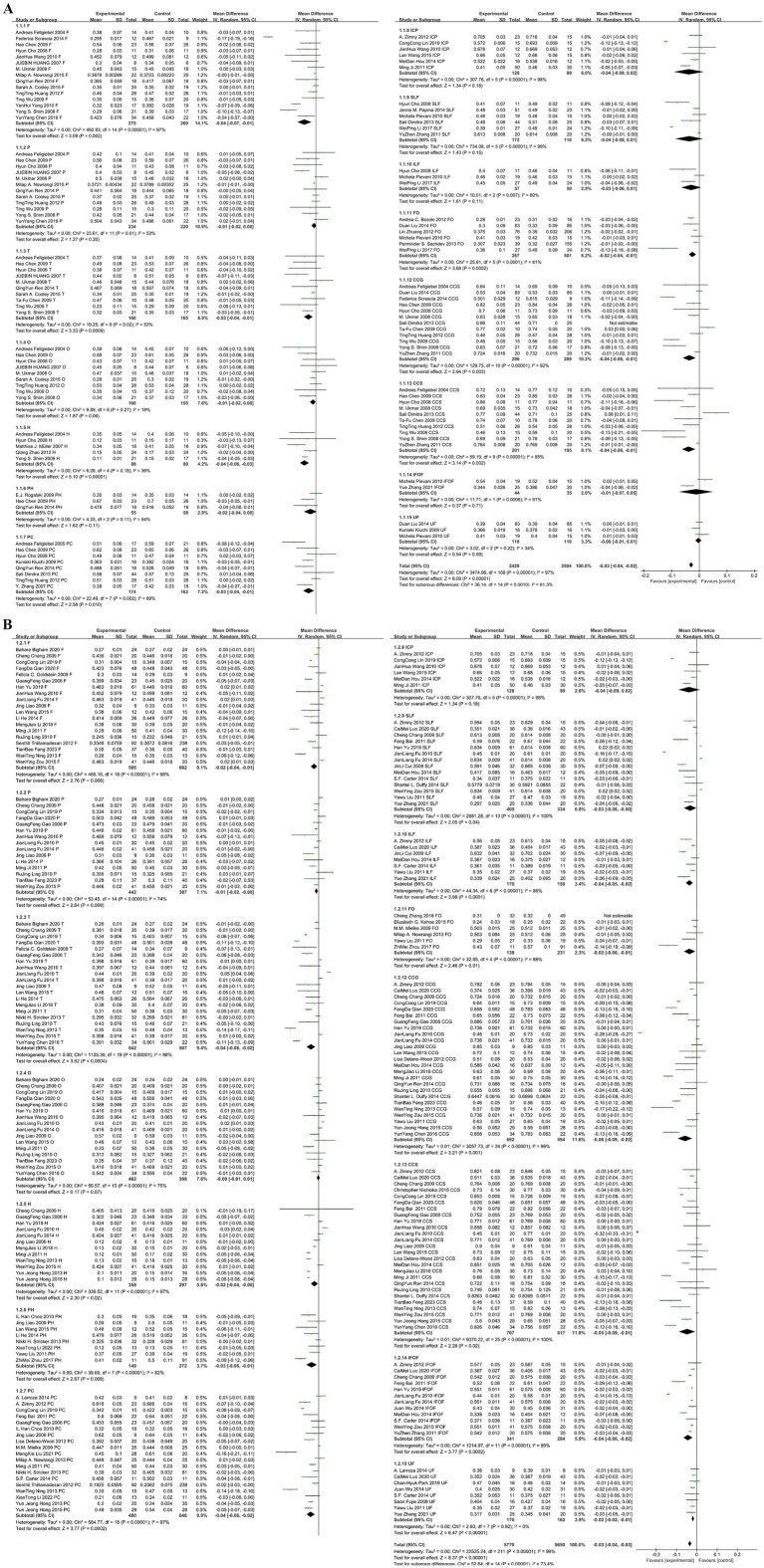
**(A)** Subgroup analysis based on a magnetic field strength of 1.5T. **(B)** Subgroup analysis based on a magnetic field strength of 3.0T.

The following hypotheses are made regarding the sources of heterogeneity in the study: (1) The included studies primarily consist of retrospective studies and follow-up studies with varying sample sizes, which may lead to selection and information bias. (2) Different experimental conditions were used across studies, such as variations in equipment models, scanning parameters, and post-processing methods, resulting in high heterogeneity between the studies. Specifically, in terms of processing methods, most studies used region of interest (ROI) measurements for FA values, while some used voxel-based morphometry (VBM) techniques, and others applied Tract-Based Spatial Statistics (TBSS) methods. The latter two methods can automatically and objectively observe changes in brain white matter fiber tracts, minimizing the impact of human factors. These factors contribute to the high heterogeneity observed in this paper. To explore the impact of different scanning parameters on the results, we conducted a subgroup analysis based on magnetic field strength (1.5 T vs. 3.0 T), as shown in Figure 5. The subgroup analysis revealed that the effect sizes were consistent for both 1.5 T and 3.0 T field strengths, both showing an effect size of −0.03 with similar 95% confidence intervals. The levels of heterogeneity were also similar, indicating significant heterogeneity in both cases. Therefore, magnetic field strength does not appear to be the primary factor contributing to the heterogeneity. Other factors, such as study design, sample size, and participant characteristics, may be the main contributors to the heterogeneity. Although there is still significant heterogeneity, the funnel plot symmetry test indicates a low risk of publication bias. Moreover, the large sample size partially offsets such biases, and the combined results maintain both clinical and statistical significance, making the overall findings relatively robust and reliable.

A previous meta-analysis (35) discussed the diagnostic value of DTI for MCI patients. The findings of this study are generally consistent with previous meta-analyses; however, we additionally report that the analysis of the posterior limb of the internal capsule showed no statistical significance, which differs from earlier studies. This discrepancy may be related to the insufficient sample size. This study explores the diagnostic value of DTI for MCI based on a large sample size, including 76 studies. The results show that the overall pooled WMD and its 95% CI were −0.03 [−0.04, −0.02], with FA values being lower in the MCI group compared to the NC group. This difference is statistically significant (*p* < 0.05), indicating significant microstructural damage in the white matter of MCI patients compared to cognitively normal individuals.

For the occipital lobe, the pooled WMD and its 95% CI were −0.00 [−0.01, 0.00], with the 95% CI crossing the null line, indicating no statistically significant difference (*p* = 0.33 > 0.05). Similarly, for the posterior limb of the internal capsule, the pooled WMD and its 95% CI were −0.04 [−0.08, 0.01], with the 95% CI crossing the null line, indicating no statistically significant difference (*p* = 0.12 > 0.05). This is consistent with previous findings, which show that the distribution of white matter abnormalities in MCI is uneven, primarily concentrated in regions connected by associative cortices (such as the posterior cingulate fibers, corpus callosum, temporal lobe, frontal lobe, and parietal lobe white matter). The internal capsule, related to motor functions, and the occipital visual radiations are largely unaffected, appearing later in the progression of dementia (36). Moreover, the inclusion of the posterior limb of the internal capsule in the study was limited (only 8 studies), and the small sample size may reduce statistical power, highlighting the need for future studies to expand data for further validation in this region.

In summary, this study conducted a meta-analysis based on a large sample size and multiple regions to evaluate the diagnostic value of DTI for MCI. The increased sample size and enriched data from different brain regions have improved the reliability of the meta-analysis. By assessing the integrity of white matter fiber tracts in various brain regions, DTI technology can, to some extent, predict the progression of MCI to AD. This further confirms the impact of white matter damage in the progression from MCI to AD, providing an objective basis for early diagnosis and intervention in MCI.

### 3.1 Limitations

(1) The included studies were primarily retrospective and follow-up studies, with varying sample sizes. (2) The types of studies and data processing methods in the included literature were not consistent. It is recommended that future research standardize imaging techniques and processing methods to improve the homogeneity of the studies. (3) Only a few of the included studies clearly defined MCI subtypes, and thus, no further analysis was conducted on different subtypes. The conclusions of the study may not be fully applicable to patients with varying degrees of mild cognitive impairment. Future research should systematically collect subtype data for analysis. (4) Some studies mainly focused on middle-aged and older adults, with a partial overlap in age-related cognitive decline. Future research should pay more attention to stratified sampling based on age groups.

## Data Availability

The original contributions presented in the study are included in the article/supplementary material, further inquiries can be directed to the corresponding author.
